# The accuracy of self-reported physical activity questionnaires varies with sex and body mass index

**DOI:** 10.1371/journal.pone.0256008

**Published:** 2021-08-11

**Authors:** Clare Quinlan, Ben Rattray, Disa Pryor, Joseph M. Northey, Kaarin J. Anstey, Peter Butterworth, Nicolas Cherbuin

**Affiliations:** 1 UC Research Institute for Sport and Exercise, University of Canberra, Bruce, ACT, Australia; 2 Discipline of Sport and Exercise Science, Faculty of Health, University of Canberra, Bruce, ACT, Australia; 3 Centre for Research on Ageing, Health and Wellbeing, Australian National University, Bruce, ACT, Australia; 4 School of Psychology, University of New South Wales, Bruce, NSW, Australia; 5 Neuroscience Research Australia, Bruce, NSW, Australia; Indiana University, UNITED STATES

## Abstract

**Background:**

Factors contributing to the accurate measurement of self-reported physical activity are not well understood in middle-aged adults. We investigated the associations between two self-reported surveys and objectively measured physical activity in middle-aged adults, and the influence of individual and sociodemographic factors on these associations, at different intensities utilizing an observational study design.

**Methods:**

Participants (n = 156) wore a SenseWear Armband^™^ (SWA) for a continuous seven-day period over the triceps of the left arm, to measure energy expenditure in metabolic equivalents. Participants also completed the Physical Activity Recall questionnaire (PAR) and Active Australia Survey (AAS). Associations were analyzed separately in general linear models for each intensity. The influence of individual and sociodemographic factors was assessed through moderator analyses.

**Results:**

The PAR and SWA were significantly positively associated at moderate (β = 0.68, 95% CI 0.16–1.20), vigorous (β = 0.36, 95% CI 0.20–0.53), moderate-to-vigorous physical activity (MVPA) (β = 0.52, 95% CI 0.20–0.83), and total METmins (β = 0.63, 95% CI 0.35–0.90), the AAS and SWA were associated at all intensities (moderate (β = 0.41, 95% CI 0.15–0.67), vigorous (β = 0.32, 95% CI 0.19–0.46), MVPA (β = 0.42, 95% CI 0.18–0.65) and total METmins (β = 0.62, 95% CI 0.29–0.96). A significant interaction between the PAR and sex for vigorous-intensity unveiled a weaker association in women. Both surveys tended to under-report physical activity. The largest margins of error were present at light and moderate intensities. For the PAR, participants reported over 20 hours, or 69% less light physical activity than recorded by the SWA per week. For the AAS, participants reported over 7 hours, or 38% less moderate physical activity. Compared to lighter intensities, time spent at a vigorous intensity was overreported by participants with the PAR and AAS by 91 and 43 minutes per week, respectively. The addition of Body Mass Index (BMI) resulted in non-significant interactions between the PAR and SWA for moderate-intensity, and the AAS and SWA for vigorous-intensity; a significant interaction between AAS and BMI indicated that the strength of the association differed by BMI for vigorous-intensity.

**Conclusions:**

The PAR and AAS are not equivalent to the SWA, and sex and BMI may alter the associations between the measures.

## Introduction

Regular and adequate levels of physical activity can counteract several risk factors associated with mortality, disease, mental health and wellbeing [1]. The middle-aged years (commonly considered 35–55 years [2]) may be a critical time for implementing interventions that focus on promoting health, such as physical activity. Higher midlife physical activity is associated with greater physical activity at older ages [3] and healthier ageing [4]. The accurate monitoring of physical activity in middle-aged adults is important in developing evidence-based recommendations of physical activity behaviors into older adulthood. Physical activity assessment typically utilizes self-report measures, ostensibly due to their cost-effectiveness and ease of use [5], despite their known limitations [6] and the increased availability of objective measurement tools.

In adults, self-report measures of physical activity tend to have low correlations with objective measures [7–10]. Accurately measuring physical activity using self-reported tools may be difficult as individuals cannot accurately estimate the amount and type of physical activity completed in the surveyed time, or precisely report the intensity of physical activity [11, 12]. The correlation between self-reported and objective measures of physical activity differ by the intensity of activities [6, 13, 14]^,^ and Body Mass Index (BMI) [11, 14], although inconsistently across studies. Previously, self-reported physical activity levels have been reported to differ by marital status [15–17]. However, the results are currently unclear. Some literature has suggested that married participants are more active than their single counterparts [15, 16], while other reports suggest no differences, or lower physical activity in married participants [18]. These investigations have relied upon self-reported physical activity, and the use of objective measures may help clarify the effect of marital status has on physical activity behaviors. To date, the influences of educational, chronic disease, and marital status on the association between self-reported and objectively measured physical activity have not been investigated in middle-aged adults.

In middle-aged and older-adults (ranges including those 55–65 years), correlations of ρ/κ = 0.43–0.68 have been found between self-reported measures and objectively measured physical activity [19–21]. These investigations have typically utilized pedometer (steps per day) or hip worn accelerometer measurements (counts per day) to summarize objectively measured physical activity. In a cohort of predominantly middle-aged adults, low correlations (ρ = 0.29 for men, and 0.25 for women) were found between measures of total minutes of physical activity of at least moderate-intensity assessed by self-report or accelerometry [11]. Further analysis revealed that correlations were weaker in overweight participants [11]. Contrarily, a higher correlation between self-reported an objectively measure physical activity in obese participants for moderate to vigorous physical activity (MVPA). As such, more research is needed to clarify these findings.

To address the limited knowledge of the association between self-reported and objectively measured physical activity, and the influence of sociodemographic factors on these associations in middle-aged adults, this paper aimed to: 1) Investigate the association between physical activity measured by two self-reported surveys and an objective measure of physical activity at a light-, moderate-, vigorous-, and MVPA while controlling for age, sex, and education, and; 2) Assess the influence of additional individual and sociodemographic factors, including BMI, chronic disease, and cohabitation on the agreement between self-reported and objectively measured physical activity.

## Materials and methods

Participants were from the Personality and Total Health through life (PATH) project, a longitudinal study described elsewhere [22]. Briefly, 7,485 participants were invited to participate in the project at baseline (1999–2000). Of the participants recruited at baseline, 2404 were in the ‘20+’ group, aged 20–24, and have been followed up every four years. At each data collection wave, additional questions and sub-studies have been added to investigate research questions appropriate to the cohort being interviewed [22]. This investigation focused on the PATH physical activity sub-study in the ’20+’ age group, now aged 37–43, during their fifth wave of data collection. The flow of participants through the study is displayed in S1 Fig.

One-thousand, four hundred and nine participants returned for the first stage of data collection in the fifth wave. The first stage of data collection involved the completion of an online survey, the primary component of the PATH study. The online survey consisted of questions pertaining to participant demographic details, mental and physical health, stressors, and social aspects of current life, and the Physical Activity Recall Survey (PAR) [22]. Of the 1409 participants who completed the online survey, 246 participants volunteered for the current sub-study and wore a SenseWear Armband^™^ (SWA; BodyMedia, PA, USA) for a continuous seven-day period. Participants were instructed to wear the SWA over the triceps muscle group of their left arm at all times of the day and night, except when immersing in water (e.g. showering). Participants (n = 69) with less than five valid days of SWA data (>20 h on the body each day, including two weekend days) [23], were excluded. Of the 156 participants with valid SWA, 93 completed the supplementary survey of questions specific to the participants’ current stage of life [22], which included the Active Australia Survey (AAS), added as part of the current sub-study. This investigation was approved by the Australian National University Human Research Ethics Committee, and all participants provided written informed consent (Human Ethics Protocol 2016/445).

Objectively measured and self-reported physical activity were the primary outcome measures of interest. Objectively measured physical activity was assessed using the SWA. Participants were instructed to wear the SWA over the triceps muscle group of their left arm for seven consecutive days (24 hours per day, unless immersing in water e.g. showering) with data recorded at one-minute intervals. Data were processed using SenseWear^™^ software (Pro version 8.1, BodyMedia, PA). Participant’s demographic data (age, sex, BMI, ethnicity, and smoker status) was combined with physical activity motion data, galvanic sweat response, and skin temperature data to calculate energy expenditure using the proprietary algorithm. Minute-by-minute data were coded by intensity; classified into MET-values of sedentary (< 1.50 METs), light (1.50–2.99 METs), moderate (3.00–5.99 METs), or vigorous (> 6.00 METs). Weekly total minutes (min∙week^-1^) were calculated for each intensity.

Self-reported physical activity was assessed with the PAR and the AAS. The PAR, adapted from the Whitehall II study [13], requires participants to recall the time (hours and minutes) spent engaging in light-, moderate-, or vigorous-intensity physical activity over the preceding seven-day period, as previously reported [24].

The AAS requires participants to report the frequency and weekly duration spent undertaking walking, other moderate-intensity activities (excluding walking), and vigorous physical activities that occurred for ≥ 10 minutes at a time [25]. As per the manual [25], weekly totals (min∙week^-1^) were calculated for moderate-intensity physical activity inclusive of walking, as well as vigorous-intensity, and MVPA.

Total physical activity (MET: min∙week^-1^) was also calculated by combining the duration of each physical activity intensity by its MET-value [24]. For the SWA, MET-values ≥ 1.5 were summed over the seven days to provide the metric Total physical activity (MET: min∙week^-1^). For the PAR, Total physical activity (MET: min∙week^-1^) was calculated with the formula MET: min∙week^-1^ = (1.5 x light min∙week^-1^) + (3 x moderate min∙week^-1^) + (6 x vigorous min∙week^-1^) (24, 25). For the AAS, Total physical activity (MET: min∙week^-1^) was calculated with the formula MET: min∙week^-1^ = (3 x non-vigorous min∙week^-1^) + (6 x vigorous min∙week^-1^), to correspond with the MET ranges of the AAS [25]. Sensitivity analysis revealed that applying different MET-values [19, 26] did not alter the results.

The moderating factors in the current investigation include self-reported age, sex, height, weight, (as calculated BMI), education level, chronic disease status, and cohabitation, evaluated during the primary online survey in the PATH study.

Education was assessed as the highest qualification achieved. From 11 initial categories, four education categories were created for use in subsequent analysis: completion of year 10 or equivalent, completion of year 12 or equivalent, completion of vocational training, and completion of a university degree.

Chronic disease was investigated as the risk of inactivity is higher, and perceptions of intensity different in persons with chronic disease [27]. Participants were asked if a doctor had told them that they had specific medical conditions and were subsequently classified into two categories, chronic disease present/absent. Chronic conditions included cardiovascular diseases and hypertension, cancers, diabetes, arthritis, asthma, chronic kidney conditions, reports of chronic pain, and chronic immune conditions.

It is unclear if marital status impacts self-reported physical activity in middle-aged adults. From four initial categories, two categories were created for subsequent analysis; those who were cohabiting with a partner, and those who were not. Including cohabitation, not just marital status accounts for non-traditional living arrangements.

Statistical analysis was conducted in R version 3.6.0. Mean and 95% confidence intervals were calculated for each measure of physical activity, subset by intensity. Adherence to the Australian Government Guidelines of 150 mins of MVPA per week [28] was assessed for each physical activity measure. Separate multivariate general linear models were computed to investigate the association between objective and self-reported physical activity measures, for each physical activity intensity. For each model, the SWA measure was the dependent variable, and the independent variable was the PAR or AAS measure, with the covariates age, sex, and self-reported level of education. Age was mean-centered at 40.6 years for the PAR and 40.4 years for the AAS. The interaction terms between the independent variable and covariates were included in the initial models. A significant interaction term indicated that that covariate may moderate the association between the SWA and PAR or AAS. Where appropriate, independent samples t-tests were conducted on significant interactions. Non-significant interaction terms were dropped from the final model for ease of interpretation. QQ-plots were generated to allow visual inspection of the residuals. All plots showed acceptable distribution.

To investigate the influence of BMI, chronic disease, and cohabitation, the above models were repeated, with each moderating factor added individually. BMI was mean-centered at 26.6 kg/m^2^ for the PAR and 26.3 kg/m^2^ for the AAS. To reduce the risk of Type I errors, a Simes-Benjamini-Hochberg false discovery rate adjustment was applied to the p-values generated from the general linear models. Statistical significance was set at adjusted p < 0.05.

Separate univariate general models were also completed to determine the association between comparable intensities for the two self-reported surveys for participants who completed both surveys, with the covariates age, sex, and education.

## Results

One hundred and fifty-six participants (58.3% female), aged 38–43 (mean 40.6 years) successfully wore the SWA and completed the PAR were included in the analysis. Of these, 93 also completed the AAS (60.2% female) aged 38–43 (mean 40.4 years). The participants with valid SWA data were older than the wider PATH study population who completed the online survey (40.6 ± 1.5 years; vs. 40.4 y ± 1.5 years p = 0.03) but were similar in the proportion of women (58.3% vs 55.5%, p = 0.50) and completion of a higher degree at university (12.2% vs 12.3%, p = 0.97).

Participants were considered most active according to the SWA, with 91% meeting the recommended guidelines of 150 minutes of MVPA per week, compared to 85% for the PAR and 73% for the AAS (Table 1).

**Table 1:** Weekly totals as assessed by the SWA, the PAR, and the AAS for the study sample. Data are mean and 95% CI.

	SWA (n = 156)	PAR (n = 156)	SWA (n = 93)	AAS (n = 93)
Light PA (min∙week^-1^)	1727 (1628 to 1825)	420 (351 to 490)	1720 (1756 to 1798)	N/A
Moderate PA (min∙week^-1^)	717 (634 to 800)	196 (173 to 220)	731 (625 to 838)	270 (194 to 346) ^a^
Vigorous PA (min∙week^-1^)	68 (51 to 85)	159 (129 to 189)	82 (56 to 108)	125 (89 to 161)
MVPA (min∙week^-1^)	785 (695 to 874)	355 (313 to 397)	813 (696 to 931)	395 (302 to 488)
Total PA (MET: min∙week^-1^)	6742 (6252 to 7233)	2171(1912 to 2429)	6863 (6206 to 7519)	1561 (1205 to 1917)
>150 mins MVPA, n (%)	142 (91)	133 (85)	85 (91)	68 (73)

N/A: AAS does not measure light-intensity PA

^a^AAS moderate-intensity PA is calculated as the sum of walking and moderate-intensity PA.

Notes: SWA: SenseWear™ Armband; PAR: Physical Activity Recall questionnaire; AAS: Active Australia Survey; MVPA: moderate to vigorous physical activity; PA: physical activity.

The analysis of the associations between the PAR and the SWA showed significant absolute differences between the PAR and the SWA at all outcomes except for vigorous-intensity, indicated by significant intercepts. Significant positive associations between the PAR and the SWA-derived physical activity were observed for all outcomes except for light-intensity. However, the adjusted R2 were small (0.13–0.20 for significant associations), indicating that a small portion of variance was explained (Table 2). There was a significant interaction between the PAR and sex for the vigorous-intensity, indicating that the association between the PAR and SWA-derived physical activity was weaker in women (t = -21.16 p <0.001). All other interaction terms were non-significant and dropped from the final models.

**Table 2:** Summary of multivariate models examining the association between physical activity as measured by the PAR questionnaire and the SWA.

	Light		Moderate		Vigorous		MVPA		Total PA (MET: min)	
	b (SE)	p^#^	b (SE)	p^#^	b (SE)	p^#^	b (SE)	p^#^	b (SE)	p^#^
Sex^	163.30 (100.62)	0.19	-352.87 (81.42)	**<0.001**	5.33 (23.93)	0.92	-376.39 (87.37)	**<0.001**	-1564.88 (470.84)	**0.003**
Age	-63.77 (31.95)	0.11	-6.61 (25.82)	0.80	-5.02 (5.21)	0.79	-7.78 (27.50)	0.78	-136.17 (148.39)	0.50
Education*	333.26 (223.34)	0.19	134.52 (180.53)	0.64	19.28 (36.19)	0.79	159.47 (191.42)	0.69	1336.34 (1034.27)	0.35
PAR*Sex	-	-	-	**-**	-0.28 (0.10)	**0.02**	-	-	-	-
PAR	0.24 (0.11)	0.11	0.68 (0.26)	**0.03**	0.36 (0.08)	**<0.001**	0.52 (0.16)	**0.003**	0.63 (0.14)	**<0.001**
Intercept	1253.63 (299.25)	**<0.001**	660.00 (188.74)	**0.002**	25.10 (39.87)	0.79	680.26 (203.16)	**0.003**	5229.15 (1092.64)	**<0.001**
Model	F6,149 = 2.45; p = 0.03; R^2^ = 0.05		F6,149 = 4.89; p = <0.001; R^2^ = 0.13		F7,148 = 6.38; p = <0.001; R^2^ = 0.20		F6,149 = 6.14; p = <0.001; R^2^ = 0.17		F6,149 = 6.93; p = <0.001; R^2^ = 0.19	

MVPA: moderate to vigorous physical activity; PA: physical activity; b, regression coefficient; SE, standard error

# adjusted for multiple comparisons

^ women compared to men (reference level: men)

*high school certificate compared to university.

R^2^ is adjusted.

The moderator analysis revealed that when BMI was added to the models, the associations of all outcomes remained unchanged between the PAR and SWA-derived measures, except for moderate-intensity, where the association was no longer significant (p = 0.11) (S1 Table). The addition of chronic disease status only altered the associations for the light-intensity outcome for the PAR; there was no longer a significant absolute difference between measures (p = 0.06) (S2 Table). The addition of cohabitation did not alter the associations within any models (S3 Table).

The analysis of the associations between the AAS and SWA also showed significant absolute difference between the measures at all outcomes except for vigorous-intensity. The multivariate models comparing the AAS to the SWA-derived variables (Table 3) showed associations for all outcomes, with adjusted R^2^ values of 0.22–0.31.

**Table 3:** Summary of multivariate models examining the association between physical activity as measured by the AAS questionnaire and the SWA.

	Moderate^a^		Vigorous		MVPA		Total PA (MET: min)	
	b (SE)	p^#^	b (SE)	p^#^	b (SE)	p^#^	b (SE)	p^#^
Sex^	-414.20 (98.88)	**<0.001**	-57.69 (23.03)	0.05	-465.26 (104.94)	**<0.001**	-2454.36 (584.38)	**<0.001**
Age	-14.70 (30.17)	0.88	-13.33 (6.80)	0.12	-27.97 (32.02)	0.67	-329.73 (175.18)	0.11
Education*	44.03 (273.09)	0.93	74.61 (64.01)	0.43	140.22 (293.66)	0.81	724.26 (1623.15)	0.77
AAS	0.41 (0.13)	**0.007**	0.32 (0.07)	**<0.001**	0.41 (0.12)	**0.002**	0.62 (0.17)	**<0.001**
Intercept	861.34 (280.45)	**0.007**	25.00 (68.00)	0.83	848.61 (308.72)	**0.02**	6982.96 (1725.79)	**<0.001**
Model	F6,86 = 5.20; p = <0.001; R^2^ = 0.22		F6,86 = 7.82; p = <0.001; R^2^ = 0.31		F6,86 = 6.60; p <0.001; R^2^ = 0.27		F6,86 = 7.38; p = <0.001; R^2^ = 0.29	

^a^ AAS moderate-intensity PA is calculated as the sum of walking and moderate -intensity PA; MVPA: moderate to vigorous physical activity; PA: physical activity; b, regression coefficient; SE, standard error

# adjusted for multiple comparisons

^ women compared to men (reference level: men)

*high school certificate compared to university.

R^2^ is adjusted.

The addition of BMI to the models created an interaction between both the AAS and BMI (p = 0.002) and education (p = 0.005) and resulted in the association between the AAS and the SWA-derived outcomes becoming non-significant for the vigorous-intensity (S4 Table). The addition of chronic disease and cohabitation did not alter the associations within the models for any outcome (S5 and S6 Tables).

Analysis revealed that the AAS and PAR were associated at all comparable outcomes (S7 Table).

## Discussion

This investigation examined the ability of middle-aged adults to accurately self-report the duration and intensity of physical activity using the PAR and the AAS. The AAS was associated with the SWA at all intensities, while the PAR was associated at all intensities except for light. Comparison of the models in Table 3 suggests that the AAS may perform better, explaining a larger proportion of the variance, although interpretation is cautious, as data for both surveys were not available for all participants. At best, the initial self-report models explained 31% of the variance in objectively measured physical activity and absolute differences were large (mean of 25–1254 mins across intensities), with self-report measures significantly under-reporting objective physical activity for all but vigorous-intensity. The predictive potential of all models increased with the addition of BMI, which also impacted the associations between measures at moderate-intensity for the PAR and vigorous-intensity for the AAS. These results emphasize the potential caveats in using self-reported tools and the associated caution required for result interpretation.

Both surveys showed several associations with the SWA, with the AAS associated at all intensities assessed. The PAR was not associated with the SWA for light-intensity and participants under-reported light-intensity by ~ 20 hours compared to the SWA over the week. Light intensity was not assessed by the AAS. If light-intensity physical activity does impact health outcomes [29], this discrepancy may have implications for public health recommendations that utilize similar self-report measures. The less structured nature and potentially shorter duration of physical activity at lower intensities (e.g. walking to get coffee, active transport) may make it more difficult to recall than vigorous-intensity [12], negatively impacting reporting accuracy. To address the call for research into the contribution of light-intensity physical activity on health outcomes [1], the use of objective measurements which capture light-intensity should be prioritized.

Despite the observed associations between the self-reported measures and the SWA at most intensities, the initial models were weak, explaining only a small proportion of variance in physical activity measured by the SWA. The weak associations between measures were paralleled by significant absolute differences between the measures across most intensities, with a tendency for under-reporting. This under-reporting meant that the discrepancies in the number of individuals achieving the recommended physical activity guidelines of at least 150 mins of MVPA per week differed by up to 18% depending on the measure utilized. When the average male within the current cohort reported 150 minutes of MVPA on the PAR (40.6 years, university educated), the SWA collected over four times the amount of MVPA. While less extreme, when 150 minutes was reported by the average male using the AAS (40.4 years, university educated), the SWA still measured double the minutes of MVPA. Only 22–32% of total physical activity, expressed in MET: min∙week^-1^, measured by the SWA was typically reported using self-report, indicating a significant proportion of total physical activity was unaccounted for when using the self-report measures. While this may appear positive, because participants were more active than they believed, this is likely problematic, as physical activity guidelines aimed at promoting health and mitigation of disease risks are typically based on self-report epidemiological data [30]. Previously a self-reported measure of physical activity underestimated the relationships between physical activity and health risk factors compared to objective measures [31]. If the observed differences between self-report and objective physical activity hold more broadly, the true activity requirements for health benefits are likely to be higher than the current recommendations. Understanding the true activity requirements, and altering physical activity behaviors in middle-age, may be particularly pertinent as physical activity behaviors and health outcomes at middle-age influence physical activity behaviors that occur into older-adulthood [3].

Consideration to the type of self-reported tool is also highlighted by the reported differences between measures. The AAS asks participants to recall physical activity that has occurred in bouts of at least 10 minutes [25]. Along with the increasing importance of physical activity intensity, so too is the importance of short bouts (<10 mins) of MVPA on healthy ageing [32], with just 10 minutes of cumulative MVPA per day associated with greater cognitive function in adults [33]. This may also be an important consideration when determining which tool is most appropriate for a given population. Compared to those previously reported, the current cohort was more physically active according to both self-reported and objective measures [19–21], however, as the studies have utilized as variety of tools, in differing populations, direct comparison is difficult. As recall is typically more difficult for brief sporadic physical activity of low intensity, those with higher levels of non-exercise activity may have a harder time accurately recalling their activity [34, 35].

Individual differences such as sex [11, 14], fitness, perceived exertion, and past physical activity experience may contribute to errors in self-reported physical activity [6], contributing to the differences observed between measures. The current results support previous findings that the association between measures of physical activity vary by sex [11, 14], potentially occurring as men typically participate in more organized sports than women [36]. The narrow age range and generally high educational status in the cohort studied may account for why neither age nor education altered the initial models. Future research expanding on the influence of specific chronic disease groups on physical activity behaviors and reporting is required, as recent accelerometer data has been observed to differ by chronic disease type, compared to those without a chronic disease [37].

This investigation found that after the addition of BMI, the PAR was no longer associated with SWA-derived moderate-intensity physical activity. For vigorous-intensity using the AAS, those with a lower BMI were better able to estimate their physical activity. Although this effect appeared small (β = -0.05), there was also a loss of an association between the AAS and SWA in the model, perhaps indicating a tenuous ability for this self-report method to predict objective physical activity. Previously, correlations between objective and self-reported physical activity have been lower for those with a higher BMI, however the impact of BMI has differed by intensity and sex [6, 11, 14]. Further, in a recent investigation [10] it was found that the correlation was higher in obese participants. The ability of overweight and obese persons to accurately self-report intensity may be reduced, as they may be working at a higher percentage of their peak capacity [27]. Alternatively, their memory of physical activity may be better due to the limited amount they are performing.

Considering the self-reported measures themselves, although associated at moderate-, vigorous-, and MVPA-intensity brackets, the measures were not comparable at light-intensity, or MET: min∙week^-1^ due to design differences. Transforming physical activity to MET: min∙week^-1^ resulted in stronger correlations with the SWA. Although the MET: min∙week^-1^ variable was less vulnerable to the addition of moderating factors, it still displayed a significant absolute difference to the SWA-derived MET: min∙week^-1^.

This study had many strengths but also a number of limitations. While the PATH study randomly samples from the ACT population, and the participants included in this sub-study were not substantially different from the cohort they were selected from, they may not be completely representative of the broader population. The results of the current investigation, although providing insight into physical activity measurement in middle-aged adults, may not be applicable to the wider population due to changing physical activity levels and reporting ability in ageing. Future research should clarify the associations between measures when controlling for sociodemographic factors, while also investigating other self-report methods. Future research may also benefit from the investigation of the influence of different chronic disease status, as different disease states will likely influence physical activity behaviors differently [37]. Although the SWA has been validated in adults [38], it is still an estimate of physical activity, and may underestimate MVPA by 2.9% [38]. However, as a large amount of physical activity undertaken by middle-aged adults is of light- to moderate-intensity, the SWA may be a more appropriate objective measure than accelerometers, as it is more accurate than accelerometry-based monitors [39]. Objective measures of physical activity become more efficacious for epidemiological research as physical activity monitoring technology continues to advance, becoming both more accurate and cost-effective. Utilizing both objective and self-report measures of physical activity may limit the misreporting of physical activity through self-report tools, and provide complimentary information that characterizes the types and perceptions of physical activity, which technology is currently less able to monitor.

## Conclusion

The use of objective measurements should be prioritized within research, particularly in samples where measurement noise may be loud. Where objective measures are not feasible, validated questionnaires should be implemented, with the choice of measure considering individual and sociodemographic factors such as sex and BMI. Consideration to continue to utilize self-report tools should also be considered, as they are often able to collect data surrounding activity type, perception of intensity, and enjoyment levels that objective measurement tools are unable to capture. These results suggest that accounting for individual and sociodemographic factors, proves difficult, and, may not improve self-reported measures to be accurately reflective of objective measurements. Studies utilizing self-reported measures should be interpreted cautiously, particularly when informing public health policy. As the self-reported measures tended to underreport physical activity significantly, a review of the physical activity guidelines may be appropriate.

## Supporting information

S1 Fig Flow of participants through investigation. SWA: SenseWear Armband™; PAR: Physical Activity Recall Survey; AAS: Active Australia Survey.
(DOCX)

S1 Table Summary of multivariate models examining the association between physical activity as measured by the physical activity recall questionnaire and the SenseWear Armband™ with BMI as a moderating factor. (DOCX)

S2 Table Summary of multivariate models examining the association between physical activity as measured by the physical activity recall questionnaire and the SenseWear Armband™ with chronic disease as a moderating factor. (DOCX)

S3 Table Summary of multivariate models examining the association between physical activity as measured by the physical activity recall questionnaire and the SenseWear Armband™ with cohabitation as a moderating factor. (DOCX)

S4 Table Summary of multivariate models examining the association between physical activity as measured by the active Australia survey and the SenseWear Armband™ with BMI as a moderating factor. (DOCX)

S5 Table Summary of multivariate models examining the association between physical activity as measured by the active Australia survey and SenseWear Armband™ with chronic disease as a moderating factor. (DOCX)

S6 Table Summary of multivariate models examining the association between physical activity as measured by the active Australia survey and the Sensewear Armband™ with cohabitation as a moderating factor. (DOCX)

S7 Table Summary of multivariate models examining the association between physical activity as measured by the active Australia survey and the physical activity recall survey. (DOCX)
