# Supplementary material for: The accuracy of self-reported physical activity questionnaires varies with sex and body mass index
Source: PLoS One. 2021 Aug 11;16(8):e0256008. doi: 10.1371/journal.pone.0256008 (PMC8357091; doi:10.1371/journal.pone.0256008)
Supplement: S1 Fig — SWA: SenseWear Armband™; PAR: Physical Activity Recall Survey; AAS: Active Australia Survey. (DOCX) [file pone.0256008.s001.docx]

S1 Fig. Flow of participants through investigation. SWA: SenseWear Armband^™^; PAR: Physical Activity Recall Survey; AAS: Active Australia Survey.

Participants interested in participation in sub study n = 246

Participants completed face to face interview n = 1259

Participants with valid SWA n = 160

Participants returned for testing, who completed primary online survey n = 1409

Participants included in analysis with valid AAS and SWA n = 93

N = 158

Participants included in analysis with valid PAR and SWA n = 156

N = 158

Participants removed due to epilepsy (n = 2)

Participants removed due to stroke (n=1)

Participant removed due to undisclosed medical history (n = 1)

Less than 5 days wear time (n = 69)

Data warning error (n = 6)

Collection error (n = 11)

Participants randomly selected at baseline n = 2404

Inclusive of BIS/BAS scales, and Goldberg questionnaire
