# Supplementary material for: The accuracy of self-reported physical activity questionnaires varies with sex and body mass index
Source: PLoS One. 2021 Aug 11;16(8):e0256008. doi: 10.1371/journal.pone.0256008 (PMC8357091; doi:10.1371/journal.pone.0256008)
Supplement: S1 Table — (DOCX) [file pone.0256008.s002.docx]

|  | **Light** | | **Moderate** | | **Vigorous** | | **MVPA** | | **Total PA (MET: min)** | |
| --- | --- | --- | --- | --- | --- | --- | --- | --- | --- | --- |
|  | b (SE) | p^#^ | b (SE) | p^#^ | b (SE) | p^#^ | b (SE) | p^#^ | b (SE) | p^#^ |
| Sex^ | 157.52 (90.20) | 0.11 | -359.49 (77.24) | **<0.001** | -47.08 (16.38) | **0.01** | -392.51 (82.11) | **<0.001** | -1663.79 (417.45) | **<0.001** |
| Age | -59.94 (28.65) | 0.10 | -5.02 (24.49) | 0.84 | -4.96 (5.14) | 0.54 | -7.49 (25.82) | 0.77 | -130.23 (131.48) | 0.37 |
| Education* | 256.14 (200.59) | 0.23 | 93.61 (171.50) | 0.67 | 15.25 (35.80) | 0.80 | 111.50 (180.04) | 0.63 | 988.30 (917.95) | 0.37 |
| BMI | -48.51 (7.93) | **<0.001** | -28.78 (6.85) | **<0.001** | -5.02 (1.43) | **0.005** | -33.19 (7.24) | **<0.001** | -237.88 (36.79) | **<0.001** |
| PAR | 0.18 (0.10) | 0.11 | 0.50 (0.25) | 0.11 | 0.14 (0.04) | **0.007** | 0.37 (0.15) | **0.03** | 0.47 (0.13) | **<0.001** |
| Intercept | 1282.43 (205.54) | **<0.001** | 699.39 (179.25) | **<0.001** | 68.62 (37.27) | 0.14 | 741.92 (191.23) | **<0.001** | 5617.76 (969.95) | **<0.001** |
| Model | F7,148=7.96; p <0.001;  R^2^=0.24 | | F7,148 =7.18; p <0.001;  R^2^=0.22 | | F7,148 =7.07; p <0.001;  R^2^=0.22 | | F7,148 =8.97; p <0.001;  R^2^=0.26 | | F7,148 =13.54; p <0.001;  R^2^=0.36 | |
| MVPA: moderate to vigorous physical activity; PA: physical activity; PAR: Physical Activity Recall questionnaire; b: regression coefficient; SE: standard error; # adjusted for multiple comparisons; ^women compared to men (reference level: men); *high school certificate compared to university | | | | | | | | | | |

S1 Table. Summary of multivariate models examining the association between physical activity as measured by the Physical Activity Recall questionnaire and the SenseWear Armband™ with BMI as a moderating factor.
