# Supplementary material for: The accuracy of self-reported physical activity questionnaires varies with sex and body mass index
Source: PLoS One. 2021 Aug 11;16(8):e0256008. doi: 10.1371/journal.pone.0256008 (PMC8357091; doi:10.1371/journal.pone.0256008)
Supplement: S2 Table — (DOCX) [file pone.0256008.s003.docx]

|  | **Light** | | **Moderate** | | **Vigorous** | | **MVPA** | | **Total PA (MET: min)** | |
| --- | --- | --- | --- | --- | --- | --- | --- | --- | --- | --- |
|  | b (SE) | p^#^ | b (SE) | p^#^ | b (SE) | p^#^ | b (SE) | p^#^ | b (SE) | p^#^ |
| Sex^ | 223.70 (97.99) | 0.05 | -359.73 (81.65) | **<0.001** | 19.61 (24.27) | 0.93 | -381.94 (87.14) | **<0.001** | -1588.15 (469.58) | **0.002** |
| Age | -61.74 (30.93) | 0.06 | -5.53 (25.83) | 0.83 | -4.53 (5.13) | 0.93 | -5.67 (27.43) | 0.84 | -127.20 (147.90) | 0.52 |
| Education* | 989.62 (391.68) | 0.05 | 122.81 (180.97) | 0.77 | 17.42 (35.73) | 0.93 | 148.89 (191.20) | 0.72 | 1227.14 (1032.37) | 0.38 |
| Chronic Disease | -341.15 (94.70) | **0.005** | -44.72 (79.62) | 0.77 | 1.62 (15.82) | 0.93 | -29.00 (84.33) | 0.84 | -612.76 (457.10) | 0.36 |
| PAR*Sex |  |  |  |  | -0.37 (0.10) | **0.002** |  |  |  |  |
| PAR*Education | -2.30 (1.07) | 0.05 |  |  |  |  |  |  |  |  |
| PAR | 2.41 (1.06) | 0.05 | 0.77 (0.27) | **0.01** | 0.46 (0.09) | **<0.001** | 0.60 (0.17) | **0.002** | 0.65 (0.14) | **<0.001** |
| Intercept | 768.98 (397.45) | 0.06 | 678.64 (195.14) | **0.003** | 9.48 (40.67) | 0.93 | 677.05 (210.10) | **0.004** | 5584.86 (1132.30) | **<0.001** |
| Model | F10,144=3.42; p<0.001;  R^2^=0.14 | | F7,147=4.48; p <0.001;  R^2^=0.14 | | F8,146=6.55; p <0.001;  R^2^=0.22 | | F7,147=5.73; p <0.001;  R^2^=0.18 | | F7,147=6.44; p <0.001;  R^2^=0.20 | |
| MVPA: moderate to vigorous physical activity; PA: physical activity; AAS: Active Australia Survey; b: regression coefficient; SE: standard error; # adjusted for multiple comparisons;  ^ women compared to men (reference level: men); *high school certificate compared to university ; Chronic disease – compared to those without a chronic disease | | | | | | | | | | |

S2 Table. Summary of multivariate models examining the association between physical activity as measured by the Physical Activity Recall questionnaire and the SenseWear Armband™ with chronic disease as a moderating factor.
