# Supplementary material for: The accuracy of self-reported physical activity questionnaires varies with sex and body mass index
Source: PLoS One. 2021 Aug 11;16(8):e0256008. doi: 10.1371/journal.pone.0256008 (PMC8357091; doi:10.1371/journal.pone.0256008)
Supplement: S3 Table — (DOCX) [file pone.0256008.s004.docx]

|  | **Light** | | **Moderate** | | **Vigorous** | |  | **MVPA** | | **Total PA (MET: min)** | |
| --- | --- | --- | --- | --- | --- | --- | --- | --- | --- | --- | --- |
|  | b (SE) | p^#^ | b (SE) | p^#^ | b (SE) | p^#^ |  | b (SE) | p^#^ | b (SE) | p^#^ |
| Sex^ | 168.45 (99.49) | 0.15 | -350.40 (81.29) | **<0.001** | -44.76 (17.03) | **0.04** |  | -373.22 (87.17) | **<0.001** | -1544.77 (466.73) | **0.003** |
| Age | -60.02 (31.63) | 0.12 | -4.84 (25.81) | 0.85 | -4.92 (5.36) | 0.63 |  | -5.72 (27.47) | 0.84 | -120.98 (147.27) | 0.55 |
| Education* | 221.79 (226.93) | 0.44 | 80.23 (185.31) | 0.85 | 19.07 (38.24) | 0.72 |  | 97.51 (196.43) | 0.84 | 861.35 (1054.24) | 0.55 |
| Relationship^~^ | -255.13 (120.28) | 0.12 | -122.56 (97.71) | 0.42 | -9.79 (20.20) | 0.72 |  | -138.85 (103.60) | 0.36 | -1071.46 (556.28) | 0.11 |
| PAR | 0.22 (0.11) | 0.12 | 0.68 (0.26) | **0.03** | 0.16 (0.04) | **0.003** |  | 0.52 (0.16) | **0.004** | 0.62 (0.14) | **<0.001** |
| Intercept | 1414.26 (238.92) | **<0.001** | 735.09  (197.66) | **0.001** | 68.73 (40.53) | 0.25 |  | 763.13 (211.85) | **0.002** | 5887.48 (1135.50) | **<0.001** |
| Model | F7,148=2.79; p =0.009;  R^2^=0.07 | | F7,148=4.43; p <0.001;  R^2^=0.13 | | F7,148=4.93; p <0.001;  R^2^=0.15 | |  | F7,148=5.55; p <0.001;  R^2^=0.17 | | F7,148=6.58; p <0.001;  R^2^=0.20 | |
| MVPA: moderate to vigorous physical activity; PA: physical activity; PAR: Physical Activity Recall questionnaire; b: regression coefficient; SE: standard error. # adjusted for multiple comparisons; ^ women compared to men (reference level: men); * high school certificate compared to university;  ^~^compared to those living with a partner. | | | | | | | | | | | |

S3 Table. Summary of multivariate models examining the association between physical activity as measured by the Physical Activity Recall questionnaire and the SenseWear Armband™ with cohabitation as a moderating factor.
