# Supplementary material for: The accuracy of self-reported physical activity questionnaires varies with sex and body mass index
Source: PLoS One. 2021 Aug 11;16(8):e0256008. doi: 10.1371/journal.pone.0256008 (PMC8357091; doi:10.1371/journal.pone.0256008)
Supplement: S4 Table — (DOCX) [file pone.0256008.s005.docx]

|  | **Moderate** ^a^ | | **Vigorous** | | **MVPA** | | **Total PA (MET: min)** | |
| --- | --- | --- | --- | --- | --- | --- | --- | --- |
|  | b (SE) | p^#^ | b (SE) | p^#^ | b (SE) | p^#^ | b (SE) | p^#^ |
| Sex^ | -389.60 (93.39) | **<0.001** | -57.81 (17.67) | **0.006** | -444.80 (98.95) | **<0.001** | -2349.47 (520.21) | **<0.001** |
| Age | -10.75 (28.44) | 0.79 | -2.80 (5.75) | 0.75 | -23.82 (29.88) | 0.57 | -297.99 (155.95) | 0.10 |
| Education* | 67.91 (257.26) | 0.79 | 11.52 (67.45) | 0.94 | 141.49 (273.86) | 0.69 | 621.02 (1443.84) | 0.76 |
| BMI | -28.25 (8.16) | **0.003** | -2.33 (1.70) | 0.42 | -32.55 (8.74) | **0.001** | -224.25 (46.05) | **<0.001** |
| AAS*Education | - | **-** | 0.53 (0.15) | **0.05** | - | - | - | - |
| AAS*BMI | - | **-** | -0.05 (0.01) | **0.002** | - | - | - | - |
| AAS | 0.33 (0.13) | **0.02** | -0.07 (0.14) | 0.75 | 0.31 (0.11) | **0.01** | 0.42 (0.16) | **0.02** |
| Intercept | 797.40 (264.74) | **0.009** | 49.91 (66.41) | 0.75 | 822.65 (287.99) | **0.01** | 6980.76 (1534.97) | **<0.001** |
| Model | F7,85=6.74; p <0.001;  R^2^=0.30 | | F11,81=13.69; p <0.001;  R^2^=0.60 | | F7,85=8.48; p <0.001;  R^2^=0.36 | | F7,85=11.38; p <0.001;  R^2^=0.44 | |
| ^a^ AAS moderate intensity PA is calculated as the sum of walking and moderate intensity PA ; MVPA: moderate to vigorous physical activity; PA: physical activity; AAS: Active Australia Survey; b: regression coefficient; SE: standard error; # adjusted for multiple comparisons; ^ women compared to men (reference level: men); *high school certificate compared to university | | | | | | | | |

S4 Table. Summary of multivariate models examining the association between physical activity as measured by the Active Australia Survey and the SenseWear Armband™ with BMI as a moderating factor.
