# Supplementary material for: The accuracy of self-reported physical activity questionnaires varies with sex and body mass index
Source: PLoS One. 2021 Aug 11;16(8):e0256008. doi: 10.1371/journal.pone.0256008 (PMC8357091; doi:10.1371/journal.pone.0256008)
Supplement: S5 Table — (DOCX) [file pone.0256008.s006.docx]

|  | **Moderate** ^a^ | | **Vigorous** | | **MVPA** | | **Total PA (MET: min)** | |
| --- | --- | --- | --- | --- | --- | --- | --- | --- |
|  | b (SE) | p^#^ | b (SE) | p^#^ | b (SE) | p^#^ | b (SE) | p^#^ |
| Sex^ | -426.00 (100.05) | **<0.001** | -58.62 (21.65) | **0.03** | -481.95 (105.48) | **<0.001** | -2495.66 (576.08) | **<0.001** |
| Age | -12.30 (30.42) | 0.87 | -11.81 (6.40) | 0.16 | -22.28 (31.89) | 0.78 | -292.11 (173.12) | 0.19 |
| Education* | 50.61 (275.22) | 0.87 | 108.35 (60.92) | 0.16 | 174.06 (292.46) | 0.78 | 1147.58 (1607.26) | 0.64 |
| Chronic disease | -16.21 (97.79) | 0.87 | -17.17 (20.80) | 0.48 | -38.42 (102.96) | 0.78 | -821.09 (560.51) | 0.23 |
| AAS | 0.43 (0.13) | **0.008** | 0.45 (0.07) | **<0.001** | 0.48 (0.12) | **<0.001** | 0.76 (0.18) | **<0.001** |
| Intercept | 866.35 (282.89) | **0.008** | -14.87 (65.09) | 0.82 | 819.99 (307.21) | **0.02** | 6784.58 (1709.77) | **<0.001** |
| Model | F7,84=4.57; p <0.001;  R^2^=0.22 | | F7,84=9.45; p <0.001;  R^2^=0.39 | | F7,84=6.24; p <0.001;  R^2^=0.29 | | F7,84=7.25; p <0.001;  R^2^=0.32 | |
| ^a^ AAS moderate intensity PA is calculated as the sum of walking and moderate intensity PA; MVPA: moderate to vigorous physical activity; PA: physical activity; AAS: Active Australia Survey; b: regression coefficient; SE: standard error; # adjusted for multiple comparisons; ^women compared to men (reference level: men); *high school certificate compared to university; Chronic disease – compared to those without a chronic disease | | | | | | | | |

S5 Table. Summary of multivariate models examining the association between physical activity as measured by the Active Australia Survey and SenseWear Armband™ with chronic disease as a moderating factor.
