# Supplementary material for: The accuracy of self-reported physical activity questionnaires varies with sex and body mass index
Source: PLoS One. 2021 Aug 11;16(8):e0256008. doi: 10.1371/journal.pone.0256008 (PMC8357091; doi:10.1371/journal.pone.0256008)
Supplement: S6 Table — (DOCX) [file pone.0256008.s007.docx]

|  | **Moderate** ^a^ | | **Vigorous** | | **MVPA** | | **Total PA (MET: min)** | |
| --- | --- | --- | --- | --- | --- | --- | --- | --- |
|  | b (SE) | p^#^ | b (SE) | p^#^ | b (SE) | p^#^ | b (SE) | p^#^ |
| Sex^ | -395.79 (99.56) | **0.001** | -55.49 (23.36) | 0.08 | -444.10 (106.58) | **<0.001** | -2330.28 (587.26) | **<0.001** |
| Age | -15.46 (30.07) | 0.71 | -13.44 (6.83) | 0.14 | -28.84 (31.87) | 0.52 | -335.05 (174.16) | 0.12 |
| Education* | -110.14 (297.45) | 0.71 | 57.04 (69.79) | 0.74 | -31.52 (318.57) | 0.92 | -266.99 (1755.46) | 0.88 |
| Relationship^~^ | -178.69 (139.33) | 0.41 | -20.60 (31.99) | 0.74 | -200.81 (148.27) | 0.36 | -1163.91 (812.52) | 0.25 |
| AAS | 0.42 (0.13) | **0.006** | 0.33 (0.07) | **<0.001** | 0.42 (0.12) | **0.002** | 0.63 (0.17) | **<0.001** |
| Intercept | 1026.03 (307.50) | **0.005** | 43.77 (74.19) | 0.74 | 1031.14 (335.49) | **0.008** | 8035.11 (1865.97) | **<0.001** |
| Model | F7,85=4.72; p =<0.001;  R^2^=0.22 | | F7,85=6.72; p =<0.001;  R^2^=0.30 | | F7,85=5.97; p =<0.001;  R^2^=0.27 | | F7,85=6.70; p =<0.001;  R^2^=0.30 | |
| ^a^ AAS moderate intensity PA is calculated as the sum of walking and moderate intensity PA; MVPA: moderate to vigorous physical activity; PA: physical activity; AAS: Active Australia Survey; b: regression coefficient; SE: standard error; # adjusted for multiple comparisons; ^ women compared to men (reference level: men); *high school certificate compared to university; ^~^compared to those living with a partner | | | | | | | | |

S6 Table. Summary of multivariate models examining the association between physical activity as measured by the Active Australia Survey and the Sensewear Armband™ with cohabitation as a moderating factor.
