# Supplementary material for: The accuracy of self-reported physical activity questionnaires varies with sex and body mass index
Source: PLoS One. 2021 Aug 11;16(8):e0256008. doi: 10.1371/journal.pone.0256008 (PMC8357091; doi:10.1371/journal.pone.0256008)
Supplement: S7 Table — (DOCX) [file pone.0256008.s008.docx]

|  | **Moderate^a^** | | **Vigorous** | | **MVPA** | |
| --- | --- | --- | --- | --- | --- | --- |
|  | b (SE) | p^#^ | b (SE) | p^#^ | b (SE) | p^#^ |
| Sex^ | -11.04 (30.95) | 0.92 | -17.74 (44.37) | 0.99 | -26.86 (51.91) | 0.86 |
| Age | -35.23 (13.09) | **0.02** | -14.26 (13.11) | 0.98 | -12.35 (15.69) | 0.86 |
| Education* | 8.54 (85.23) | 0.92 | 25.24 (123.30) | 0.99 | 26.07 (143.90) | 0.86 |
| AAS*Age | 0.12 (0.04) | **0.02** |  | **-** |  | - |
| AAS*Education* | - | **-** |  | **-** |  | - |
| AAS | 0.20 (0.06) | **0.01** | 0.61 (0.13) | **<0.001** | 0.40 (0.06) | **<0.001** |
| Intercept | 172.65 (88.09) | 0.11 | 86.89 (130.96) | 0.99 | 228.72 (151.28) | 0.47 |
| Model | F7,85 =2.24; p = 0.04;  R^2^=0.09 | | F6,86=4.64; p = <0.001;  R^2^=0.19 | | F6,86= 9.31; p = <0.001;  R^2^=0.35 | |
| ^a^ Moderate as measured by the PAR was compared to moderate (inclusive of time spent walking) for the AAS; PAR: Physical Actviity Recall survery; AAS: Active Australia Survey; MVPA: moderate to vigorous physical activity; PA: physical activity; b, regression coefficient; SE, standard error; # adjusted for multiple comparisons; ^ women compared to men (reference level: men); *high school certificate compared to university  R^2^ is adjusted | | | | | | |

S7 Table. Summary of multivariate models examining the association between physical activity as measured by the Active Australia Survey and the Physical Activity Recall survey.
